# P-07 - A REVIEW OF THE NORTHEAST PERTUSSIS CASES NOTIFIED DURING THE 2023/24 RESURGENCE: HOW DID VACCINATION STATUS AND TIME SINCE LAST DOSE IMPACT INFECTION RISK?

**DOI:** 10.1093/pubmed/fdag046.078

**Published:** 2026-07-09

**Authors:** Mrs Stephanie Baker, Dr Kate Houseman, Mr Adrian Wensley, Dr Joanne Darke, Professor Mark Pearce

**Affiliations:** UK Health Security Agency (UKHSA); UK Health Security Agency (UKHSA); UK Health Security Agency (UKHSA); UK Health Security Agency (UKHSA); Newcastle University

## Abstract

**Background:**

During the period December 2023 to December 2024, a resurgence in pertussis was seen across England. We describe the epidemiology of pertussis cases notified to the Northeast Health Protection Team (NEHPT) over this period and analyse the impact of vaccination status to identify potential recommendations for current vaccination policy.

**Methods:**

Descriptive statistics were used to characterise the study population in relation to time, place and person. Associations between risk factors and disease were determined by standard uni-variate tests.

**Results:**

During this resurgence, the NEHPT received almost 10 times the rate of pertussis notifications in children than expected. Of these, 43% were laboratory confirmed and >80% were fully immunised for their age. Of those fully vaccinated, 76.6% were laboratory confirmed. Those that were fully vaccinated were significantly more likely to be a confirmed case in the study population (p<0.001).

Cases aged 10-14 years comprised 32% of the study population and 50% of the fully vaccinated confirmed cases (p<0.001). There was no difference in the age groups across unvaccinated cases.

A significantly greater proportion of vaccinated cases had been vaccinated 5-10years prior to disease compared to 0-1 years, 1-4years and 10+years (p<0.001).

Most cases identified as being of white ethnicity and lived in the most deprived areas. Vaccination coverage was lowest in those of non-white ethnicity living in the most deprived areas.

**Discussion:**

There was a clear peak of fully vaccinated, confirmed cases reported 5-10 years after last dose of vaccine, suggesting that waning immunity was a significant driver of the recent Northeast resurgence. Further research should consider if introduction of an adolescent pertussis booster might limit future resurgences. Optimising vaccination uptake amongst those of non-white ethnicity living in the most deprived areas is required to reduce health inequalities in our population.

